# Geotemporal Fluorophore Biodistribution Mapping of Colorectal Cancer: Micro and Macroscopic Insights

**DOI:** 10.3390/curroncol31020063

**Published:** 2024-02-02

**Authors:** Niall P. Hardy, Niall Mulligan, Jeffrey Dalli, Jonathan P. Epperlein, Peter M. Neary, William Robertson, Richard Liddy, Stephen D. Thorpe, John J. Aird, Ronan A. Cahill

**Affiliations:** 1 UCD Centre for Precision Surgery, School of Medicine, UCD, D07 Y9AW Dublin, Ireland; niall.hardy@ucdconnect.ie (N.P.H.); jeffrey.dalli@ucdconnect.ie (J.D.); 2 Department of Histopathology, Mater Misericordiae University Hospital, D07 R2WY Dublin, Irelandwrobertson@mater.ie (W.R.); richard.liddy@hse.ie (R.L.); johnaird@mater.ie (J.J.A.); 3 IBM Research Europe, D15 HN66 Dublin, Ireland; jpepperlein@ie.ibm.com; 4 Department of General and Colorectal Surgery, University Hospital Waterford, University College Cork, X91 ER8E Waterford, Ireland; peter.neary@ucc.ie; 5 UCD School of Medicine, University College Dublin, D04 V1W8 Dublin, Ireland; stephen.thorpe@ucd.ie; 6 UCD Conway Institute, University College Dublin, D04 V1W8 Dublin, Ireland; 7 Department of General and Colorectal Surgery, Mater Misericordiae University Hospital, D07 R2WY Dublin, Ireland

**Keywords:** colorectal cancer, fluorescence-guided surgery, fluorescence microscopy, fluorophore biodistribution

## Abstract

Fluorescence-guided oncology promises to improve both the detection and treatment of malignancy. We sought to investigate the temporal distribution of indocyanine green (ICG), an exogenous fluorophore in human colorectal cancer. This analysis aims to enhance our understanding of ICG’s effectiveness in current tumour detection and inform potential future diagnostic and therapeutic enhancements. Methods: Fifty consenting patients undergoing treatment for suspected/confirmed colorectal neoplasia provided near infrared (NIR) video and imagery of transanally recorded and ex vivo resected rectal lesions following intravenous ICG administration (0.25 mg/kg), with a subgroup providing tissue samples for microscopic (including near infrared) analysis. Computer vision techniques detailed macroscopic ‘early’ (<15 min post ICG administration) and ‘late’ (>2 h) tissue fluorescence appearances from surgical imagery with digital NIR scanning (Licor, Lincoln, NE, USA) and from microscopic analysis (Nikon, Tokyo, Japan) undertaken by a consultant pathologist detailing tissue-level fluorescence distribution over the same time. Results: Significant intra-tumoural fluorescence heterogeneity was seen ‘early’ in malignant versus benign lesions. In all ‘early’ samples, fluorescence was predominantly within the tissue stroma, with uptake within plasma cells, blood vessels and lymphatics, but not within malignant or healthy glands. At ‘late’ stage observation, fluorescence was visualised non-uniformly within the intracellular cytoplasm of malignant tissue but not retained in benign glands. Fluorescence also accumulated within any present peritumoural inflammatory tissue. Conclusion: This study demonstrates the time course diffusion patterns of ICG through both benign and malignant tumours in vivo in human patients at both macroscopic and microscopic levels, demonstrating important cellular drivers and features of geolocalisation and how they differ longitudinally after exposure to ICG.

## 1. Introduction

Fluorescence-guided surgery is becoming increasingly utilized for perfusion assessment across many specialties, including in the management of colorectal cancer, with the next focus for field advance being cancer identification and delineation for intraoperative guidance [1,2,3,4,5,6]. New near infrared (NIR) fluorophores are in clinical trials for this purpose and potentially also for targeted cancer immunotherapy [7,8,9]. Even now, the sole approved fluorophore, indocyanine green (ICG), is being assessed for its usefulness in cancer marking in surgery for different subtypes (ICG trapping within malignant tissue has been attributed to enhanced vascular leakage, involving inflammatory mediators, increased tumour vascularity and clathrin-mediated endocytosis) and is showing promise as a means by which to characterize endoscopic cancer using computer vision and artificial intelligence methods to exploit dynamic inflow/outflow comparative perfusion/diffusion differentials between areas of neoplasia and adjacent normal tissues [10,11,12,13]. However, underlying molecular mechanisms of tumour–dye interactions (as opposed to cancer cell–dye pharmacokinetics) in humans, are poorly described. Here we examine the geo-temporal localization of ICG, the prototypical fluorophore, through initial in vivo tumoral delivery, to later tumour dispersion and then up to a timepoint of eight hours after injection to better understand tumoral distribution actors and actions and inform further studies regarding the use of fluorescence-guided cancer targeting. All patient participants had consented for recruitment into prospective clinical trials, including intraoperative ICG macroscopic tumour assessment of areas of luminal neoplasia, as well as tissue sampling for advanced basic science microscopy, in order to longitudinally compare ICG appearances with standard clinical histopathological appearances.

## 2. Methods

Fifty consenting patients with suspected/confirmed colorectal cancer undergoing theranostic procedures for primary disease in the rectum at two university hospitals provided combinations of intraoperative dynamic perfusion angiograms from their endoscopically directly observed tumours (*n* = 26, 13 cancers), fluorescence photographs of resected neoplastic specimens (*n* = 13 cancers) or tissue samples for microscopic analysis (*n* = 14, 13 cancers) as part of a larger prospective observational study (NCT04220242, approval reference number 1/378/2092).

### 2.1. Macroscopic ICG Profiling

High definition, 30-frames-per-second tumour videos were recorded following intraoperative intravenous ICG administration (0.25 mg/kg) using a near-infrared imaging system (Pinpoint, Novadaq, Stryker Corp., Kalamazoo, MI, USA) while the lesions were under direct endoscopic observation using a transanal access platform. Perfusion–diffusion profile maps of tumours were created through video stabilization (negating camera and tissue movement) extracting fluorescence values on a pixel-by-pixel basis at seven frames per second (FPS) across the full image. The image was then re-displayed in a 2D format using piecewise constant approximation of the profiles and unsupervised clustering as well as centre of mass (COM, representing the weighted average of fluorescence intensity over time with both ICG inflow and outflow rates incorporated within a single value) and outflow slopes (previously identified as a useful discriminator of malignant vs. benign tissue and therefore also included for depiction via 2D mapping) by a yellow-green-blue/viridis scale [11].

Delayed COM values (further to the right on the *x*-axis) within fluorescence curves are depicted in yellow with an earlier COM depicted in blue. ICG decay slopes are assessed between the timepoints “peak fluorescence plus 10 s” and “peak fluorescence plus 70 s”, with red indicating a negative slope and blue a positive slope upon 2D recreation. Quantitative assessment using ImageJ v1.54f (National Institutes of Health, Bethesda, MD, USA) was performed on the perfusion profiles by assessing the standard deviation and kurtosis of pixel intensity within lesions (with standard deviation of pixel brightness in an area being taken as a measure of heterogeneity of perfusion pattern within the same area), after conversion to greyscale, and comparing these by group (cancer vs. benign) using Mann–Whitney U testing (Supplementary Figure S1).

Late macroscopic appearances of malignancy (*n* = 13) were assessed using fluorescence photographs of resected neoplastic specimens and processed using ImageJ in a similar fashion. Malignant lesions were annotated, and fluorescence intensity properties (including mean intensity, maximum and minimum intensities, standard deviation, skew and kurtosis) extracted and compared with adjacent healthy tissue using Mann–Whitney U testing. Statistical analysis was performed using SPSS Statistics V.26 (IBM, Armonk, NY, USA).

### 2.2. Fluorescence Microscopy: Tissue Preparation and Analysis

Tissue samples were taken from fourteen patients for microscopic examination and for correlation with macroscopic appearances observed intraprocedurally while maintaining standard operating clinical and histopathological protocols. Tissue obtained through biopsy within 15 min of ICG administration, and during the time of direct ICG–NIR visualisation with the endoscopic NIR system, formed the ‘early’ sample group. ‘Late’ samples (>2 h post administration) were obtained from colorectal samples after radical oncological resection had been performed and the specimen extracted. Samples were obtained from both tumour and surrounding healthy mucosa, mounted in OCT, flash frozen using Lamb’s freezing aerosol and serial specimens cut using a cryotome to a thickness of 5 micrometres. One sequential sample per patient was stained with haematoxylin and eosin (H&E) to maximise contrast under white light examination and for comparison with the obtained fluorescence images. Samples for fluorescence examination were unstained as H&E has previously been shown to significantly reduce fluorescence intensity [14].

Fluorescence intensity values in slides were first assessed using a LI-COR Odyssey DLx near-infrared fluorescence imaging system. Slides with tissue samples (cancerous and healthy) were placed face down and scanned, without focus offset, at a resolution of 21 microns. Once scanned, an unstained control slide was assigned as ‘background’, each specimen was digitally outlined to obtain a ‘total intensity’ and a final signal intensity calculated (total intensity minus background). Finally, as sample sizes varied, signal intensity was divided by sample area to allow for comparison across samples. Mann–Whitney U testing was performed to compare average healthy tissue fluorescence values with cancer (where >1 sample existed per patient, values were averaged prior to analysis). Higher resolution assessment using a Nikon Eclipse Ti2 inverted research microscope was performed in twelve patient samples varying between 10×–40× magnification, and with 770 nm and 800 nm excitation and emission wavelengths, respectively. Slides were annotated by an expert pathologist’s judgement of the geographical distribution of ICG. Random fluorescence intensity sampling of specimens was also performed with eight intensity readings of “stromal intensity”, “healthy gland” and “malignant tissue” obtained per patient. Readings were divided into ‘early’ and ‘late’ samples and compared between tissue locations using Kruskal–Wallis and pairwise post-hoc analysis with Bonferroni correction.

## 3. Results

### 3.1. Macroscopic ICG Tumour Perfusion Profiling

**Figure 1:**
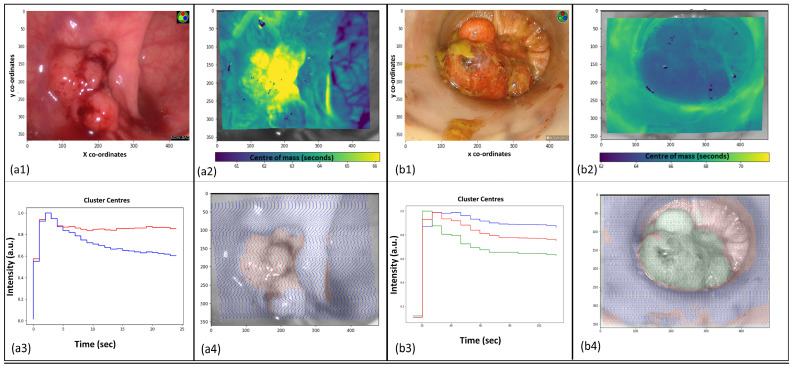
Images showing ‘early’ timepoint malignant and benign rectal lesions with white light imagery and 2D dynamic perfusion profile mapping. The outer grey borders around images represent regions lost during tracking. X and Y co-ordinates are constant across all images. (**a1**): White light view of a malignant rectal polyp. (**a2**): Pixel-by-pixel dynamic perfusion curves of malignant rectal polyp represented by a centre of mass heat map demonstrating intra-lesional heterogeneity. (**a3**): Graph representations of the two clusters (red and blue) created from assessment of time–fluorescence curves. (**a4**): Resulting heatmap following image creation using unsupervised clustering. Intralesional heterogeneity (red and blue) consistent with malignancy. (**b1**): White light view of benign rectal polyp. (**b2**): Pixel-by-pixel dynamic perfusion curves of benign rectal polyp represented by a centre of mass heat map demonstrating intra-lesional homogeneity. (**b3**)**:** Three cluster centres created from all tracked pixel data. (**b4**): Clustering was applied to create an image showing intra-lesional homogeneity.

Indicative examples of the 2D representations generated from dynamic perfusion profiles, including using unsupervised clustering, are shown in Figure 1 (with further images being presented in Supplementary Figures S2 and S3). Clustering, in particular, demonstrates heterogenous fluorescence dispersion through malignant tumours that have two different perfusion patterns within the same lesion and have relatively homogenous intralesional appearances/single clusters in benign lesions, similar to the appearances of any observable and adjacent normal tissue (Figure 1). The discrimination of created 2D rectal lesions (cancer vs. benign) and the extent of cancer examined through the assessment of intra-lesional heterogeneity of perfusion patterns (measured via standard deviation of pixel brightness) were each statistically significant and determined with ImageJ interrogation (*p* < 0.001) (see Table 1 and Supplementary Figure S1).

**Figure 2:**
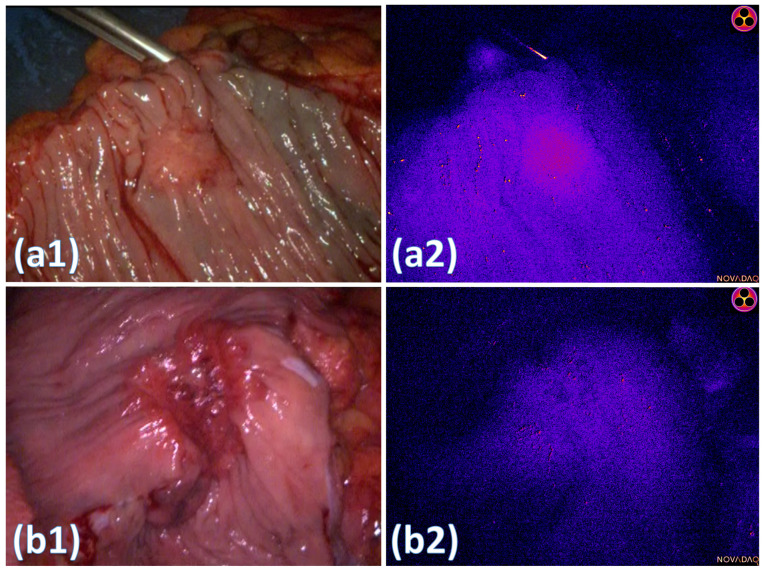
Images depicting macroscopic appearances of ‘late’ timepoint malignant lesions. White light images of malignancy are shown in (**a1**,**b1**). Fire colourmaps of fluorescence intensity show a relatively homogenous lesion demarcated from surrounding healthy tissue in (**a2**) with a less clearly demarcated lesion shown in (**b2**). (Fire colourmaps were created by converting the NIR images to 8-bit greyscale followed by application of a “fire” heatmap using a lookup table (LUT) in ImageJ).

The results of ‘late’ macroscopic cancer assessment are also shown in Table 1, with single timepoint heatmapping of two sample lesions shown in Figure 2. Though mean fluorescence intensity values were higher in malignant tissue compared with healthy regions, this did not reach statistical significance (*p* 0.153). Unlike in the ‘early’ dynamic analysis, ‘late’ analysis of intralesional heterogeneity (represented by standard deviation, skew and kurtosis) did not differ significantly from non-malignant tissue.

### 3.2. Fluorescence Microscopy: Tissue Analysis

The described tissue preparation methodology generated analysable tissue samples in all cases, with only minimal tissue folding and ‘freeze artefact’. The serial sectioning technique that was employed permitted easy identification and cross referencing of identical landmarks/tissue areas from the unstained samples to the H&E slides.

LI–COR slide scanning demonstrated a higher mean fluorescence/area score for cancer versus healthy tissue (4.593 fluorescence intensity units/area vs. 7.267), but the distribution of fluorescence between the two groups did not reach statistical significance with Mann–Whitney *U* testing (*p* = 0.436). A Kruskal–Wallis test showed significantly higher fluorescence intensities in the stroma compared with benign and malignant glands at ‘early’ timepoints (*p* < 0.001). At ‘later’ timepoints there were significant fluorescence intensity differences between malignant and benign glands (<0.001) and benign glands vs. stroma (*p* = 0.014), but not between malignant glands and stroma (*p* = 0.297).

**Figure 3:**
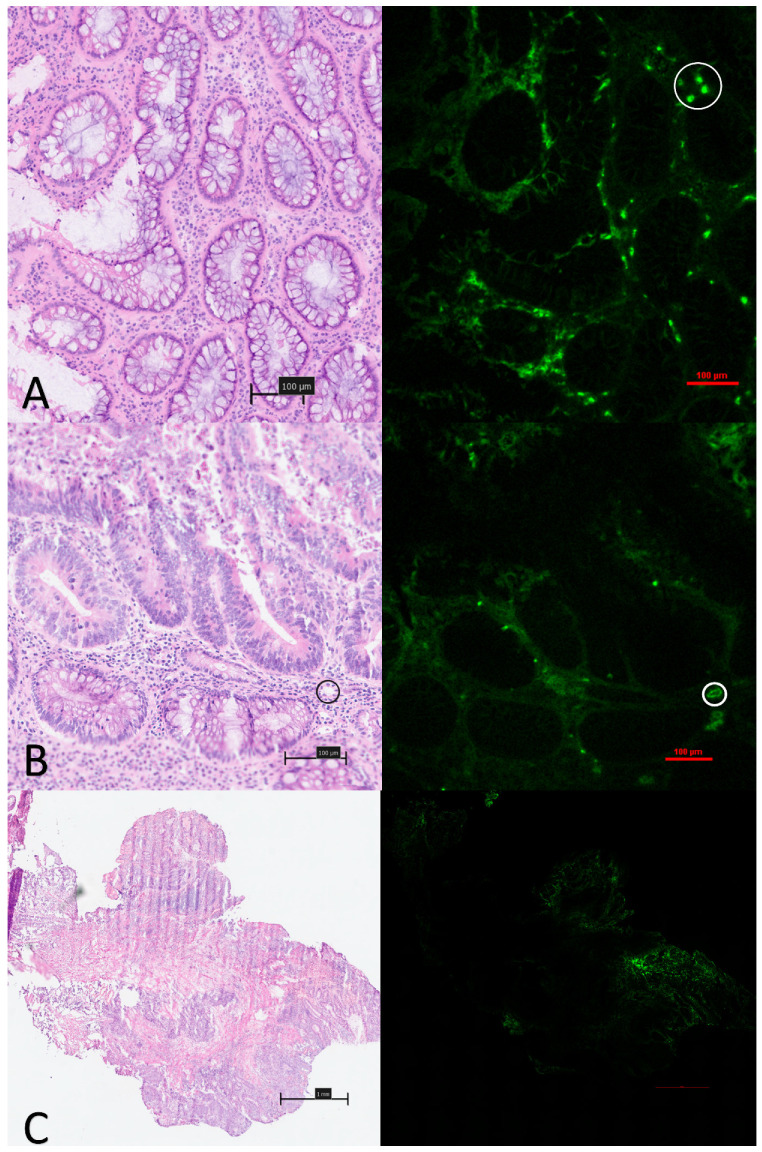
Compound white light and NIR fluorescence microscopy images of ‘early’ phase tissue samples: (**A**): 10× magnification image of relative stromal fluorescence concentration with minimal uptake seen within the glandular tissue at early timepoints. Concentration within plasma cells marked with white circle (top right). (**B**): Images taken from a low grade, poorly differentiated adenocarcinoma demonstrating a small volume of tumour with a surrounding predominantly inflammatory component in which ICG is seen to accumulate. (**C**): Images from a poorly differentiated adenocarcinoma demonstrating early strong uptake within lymphatics and vascular channels (black and white circles) but no relative uptake within abnormal glands.

Qualitative microscopic analysis demonstrated consistent patterns, which varied by time from ICG administration to biopsy acquisition. ‘Early’ samples (<15 min post ICG administration) consistently demonstrated intratumoral fluorescence intensity as a predominantly intra-stromal phenomenon with similar appearances in both normal/healthy tissue as well as in more irregular and dysplastic glands. (Figure 3A). In particular, ICG uptake within the stroma was found to be associated predominantly with plasma cell presence (white circle Figure 3A). Early presence within lymphatic channels, as well as within bloods vessels, was also noted (Figure 3B) and in one sample—a low grade, moderately differentiated, T2 lesion—a higher concentration of ICG was noted surrounding (but not within) malignant glands, compared with other non-malignant regions within the same tissue sample (Figure 3C).

**Figure 4:**
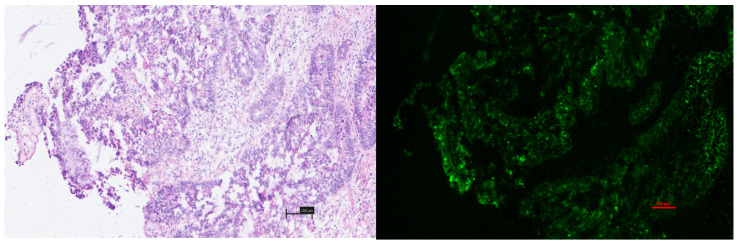
‘Late’ obtained sample of colorectal adenocarcinoma demonstrating a reversal of appearances seen at the ‘early’ timepoints, with intracellular cytoplasmic uptake of ICG within malignant glands and a relative lack of ICG within the neoplastic stroma.

‘Late’ samples (>2 h post ICG administration) consistently demonstrated the intracellular cytoplasmic accumulation of fluorescence within malignant tissue in a fashion not seen at early timepoints but was inconsistent between different malignant glands in the same sample (Figure 4). In many instances this was accompanied by a relative lack of fluorescence within the surrounding neoplastic stroma.

**Figure 5:**
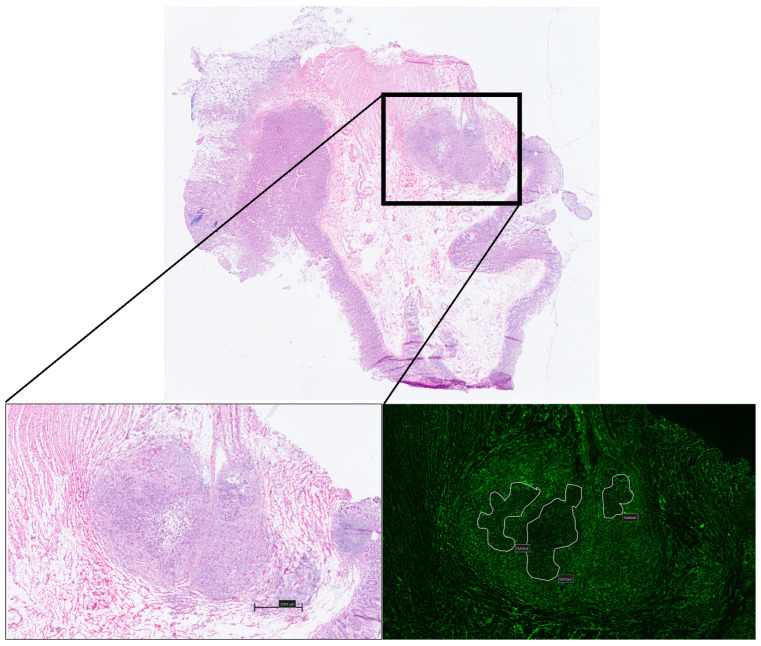
Microscopic images of a ‘late’ sample, poorly differentiated mucinous tumour with a large surrounding inflammatory component. NIR fluorescence image taken from the region marked by the black rectangle, with malignant regions marked by white outline, and with a concentration of fluorescence within surrounding inflammatory cells.

**Figure 6:**
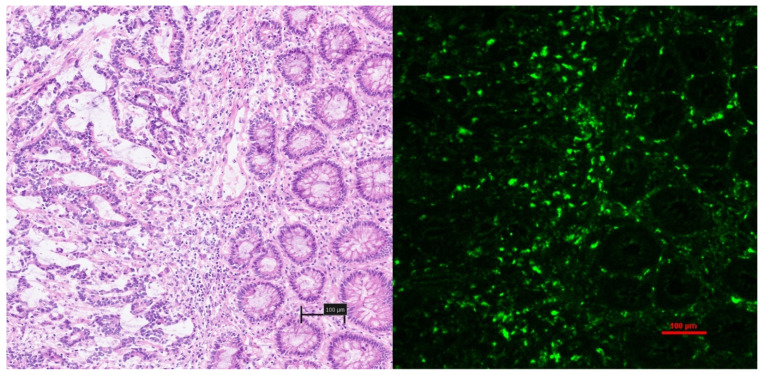
Narrower field of view from patient in Figure 5 demonstrating a concentration of ICG within the inflammatory interphase between malignant and healthy glands.

**Figure 7:**
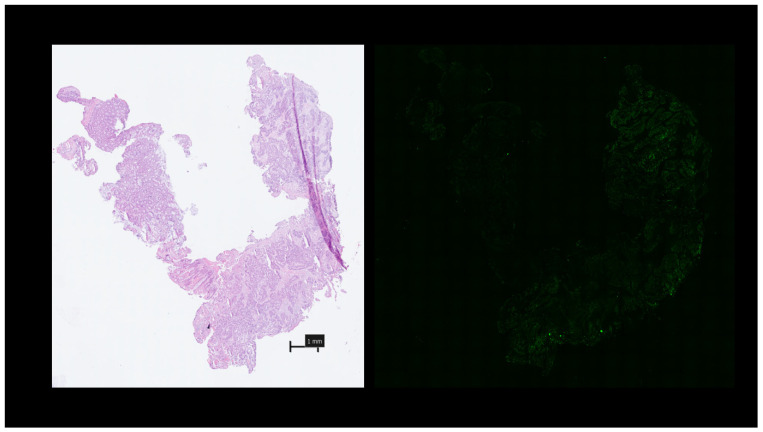
‘Late’ phase fluorescence appearances with relative concentration within malignant cells (**right and bottom**) and with washout from adjacent normal tissue (**top left**).

Figure 5 displays white light and NIR fluorescence appearances of a poorly differentiated mucinous tumour with a large surrounding inflammatory component. Though some ICG is demonstrated within the malignant tissue itself, it predominates within the immediately surrounding inflammatory component both within distinctly abnormal tissue regions as well as at areas of malignant–healthy tissue interphases (Figure 6). Where larger samples were obtained with both malignant and non-malignant areas in close proximity, higher comparative fluorescence intensities were seen within the malignant regions (Figure 7).

## 4. Discussion

The target of an oncological surgery is a tumour, which represents a complex tissue structure comprising living and dead cancer cells within a stroma of induced host reaction containing an admixture of cellular and matrix elements that favour (e.g., angiogenesis induced by the cancer) and oppose (e.g., immunoinflammatory host response) cancer development [15,16,17]. While fluorophores in development predominantly commence under laboratory examination regarding cancer cell selectivity, in clinical practice such agents need adequate permeation of the tumour infrastructure before encountering cancer cell membranes. ICG, with its excellent safety profile and low cost, remains the sole approved fluorophore for human use and it has, despite a lack of a cancer cell targets, demonstrated effectiveness in localizing cancers (including those affecting the gastrointestinal tract, gynaecological system, breast, and peritoneum, among others) [18,19,20,21,22]. This indicates that many malignant tumours demonstrate avidity for circulating substances, irrespective of cell selectively and such agents can become trapped in tumours whether at stromal or cellular levels (or a combination of both) with differential appearances happening within minutes of ICG administration.

Initial efforts involving the administration of ICG, days in advance of tissue interrogation and relying on sufficient signal–background fluorescence ratios to identify areas of malignancy, have suffered low specificity; however, when ICG persists within non-malignant areas, such as inflammation, timepoint observation as a method of cancer detection is frustrated [23]. As a result, more recent efforts have focused on early-phase dynamic tumour profiling using intraoperatively, intravenously administered ICG with comparative analysis of fluorescence in healthy and unhealthy tissue over time using computer vision and artificial intelligence, while other approaches have involved advancing cell targeted agents (such as folate-receptor targeting and tumour-vessel targeting agents such as nerve growth factor-tumour necrosis factor (NGR-TGF)) for similar effect [24,25].

‘Early’ phase macroscopic dynamic analysis demonstrated significantly more heterogeneity within malignant tissue compared with non-malignant. This dynamic, full field of view profiling may help explain the false negative biopsy results that undermine endoscopic tissue in approximately 20% of cases and is highlighted at a microvascular tissue level. It also serves as a discriminating target for tissue characterisation by characterising and analysing these findings in real time [26]. ICG outflow (represented graphically here by the downslope after peak) has been identified as an important predictor of malignancy along with curve centre of mass (COM), a function of both inflow and outflow, and both are used here to graphically represent the differences, not only to the surrounding healthy tissue, but also within different locations of a malignant lesion [11,14]. Such intra-lesional heterogeneity is not seen when the same methods are applied to benign pathologies, a finding that has been utilized to characterise rectal tumours in real time with high accuracy [20]. Microscopic interrogation suggests that these distinctions arise from variations within the neoplastic stroma and are potentially attributable to the absorption by adjacent lymphocytes. Additionally, this interrogation suggests that these distinctions form as a consequence of ICG extravasation from the notably permeable blood vessels observed in malignant tissue, rather than originating from activity at a malignant cellular level [27].

‘Late’ analysis of malignancy did not demonstrate this same level of heterogeneity macroscopically and, although malignant lesions were more fluorescent on average, this did not reach significance and is consistent with previous studies where single timepoint interrogation of malignant colorectal lesions has been shown to be of limited utility [28]. ‘Late’ microscopic analysis clearly demonstrated the intracellular uptake of ICG into colorectal cancer tissue and, to our knowledge, this is the first time that this has been demonstrated, in vivo, in humans. It is notable, however, that this was not seen ubiquitously across all cancer specimens and, indeed, that it occurred heterogeneously even within single patient samples. Inflammation also consistently contributed to fluorophore absorption, with ICG being observed to become trapped within the peritumoral inflammation, rather than penetrating into the malignant cells themselves. These findings provide insights into the intratumoural delivery of labelling and chemotherapeutic agents, with cancer representing a complex microenvironment that has an interplay between malignant and inflammatory cells and ICG, which, in this example, is able to represent any non-selective delivery agent (although even “selective” agents, proposed as potential solutions to this problem, will undoubtedly suffer from the non-selective extravasation and neoplastic stroma absorption demonstrated in this study). Further study into the role of inflammation and fluorophore uptake is warranted based on these findings, given the known association between tumour inflammation and prognosis [29,30]. The capability of NIR imaging to penetrate beyond 10 mm may allow prognostic factors such as tumour inflammatory components to be elucidated in real time and prior to tumour excision, potentially providing prognostic insights at the time of initial encounter [31].

Limitations to this study include the small number of patients included for microscopic analysis. Ideally ‘early’ and ‘late’ samples would be taken from the same patients; however, this does not fit with the clinical pathways of patients presenting for either local tumour assessment or radical excision in a single sitting. The risk of selection bias exists when randomly selecting tissue regions (stroma, healthy and malignant glands) for microscopic intensity analysis; however, the quantitative findings were in keeping with expert qualitative assessment prior to any intensity measurements being performed.

This study demonstrates the time course diffusion patterns of ICG through both benign and malignant tumours in vivo, in human patients at both macroscopic and microscopic levels, demonstrating the important drivers of geolocalisation and how they differ longitudinally after exposure to ICG. Further in-depth study is needed, building on this work, to fully characterize fluorophore location in cancer, including encompassing the dynamics of cell–cell interaction and exploring organoid models.

## Figures and Tables

**Table 1:** Intra-tumoral comparisons of ‘early’ fluorescence intensity values between malignant and benign tumours followed by ‘late’ comparison of malignant and healthy tissue. (g.u. = greyscale units). * denotes statistical significance.

Early Macroscopic Profiling	Mean Values ± Std Dev		Mann–Whitney U Testing (*p* < 0.05)
Variable	Benign Tumours (*n* = 13)	Cancerous Lesions (*n* = 13)	
Mean pixel intensity (g.u.)	102.55 ± 4.91	121.53 ± 11.25	<0.001 *
Standard deviation (g.u.)	7.85 ± 3.44	18.87 ± 6.91	<0.001 *
Mode (g.u.)	101.85 ± 3.85	135.08 ± 33.53	<0.001 *
Min (g.u.)	61.23 ± 14.74	54.46 ± 12.48	0.053
Max (g.u.)	153.31 ± 20.39	171.62 ± 7.48	0.017 *
Median (g.u.)	101.77 ± 3.59	117.38 ± 13.43	<0.001 *
Skew	−0.22 ± 1.90	0.59 ± 0.72	0.281
Kurtosis	10.61 ± 14.95	1.92 ± 3.49	0.010 *
**Late Macroscopic Profiling**	**Mean Values ± Std Dev**		**Mann–Whitney U Testing** **(*p* < 0.05)**
**Variable**	**Healthy Tissue Regions** **(*n* = 13)**	**Cancerous Tissue** **(*n* = 13)**	
Mean pixel intensity (g.u.)	29.70 ± 20.04	40.59 ± 22.18	0.153
Standard deviation (g.u.)	10.15 ± 4.31	10.82 ± 3.21	0.448
Mode (g.u.)	27.92 ± 23.31	40.15 ± 25.43	0.186
Min (g.u.)	0 ± 0	1.15 ± 3.09	0.336
Max (g.u.)	168.31 ± 78.67	164.85 ± 48.79	0.724
Median (g.u.)	29.07 ± 20.99	40.62 ± 22.59	0.139
Skew	1.62 ± 2.06	0.61 ± 1.58	0.101
Kurtosis	22.52 ± 41.29	9.63 ± 20.04	0.264

## Data Availability

Anonymised data available upon reasonable request.

## Supplementary Materials

The following supporting information can be downloaded at: https://www.mdpi.com/article/10.3390/curroncol31020063/s1, Figure S1: Analysis of mean and standard deviation of pixel intensities within lesions; Figure S2: Photographs of three malignant rectal lesions with 2D center of mass and outflow slope heatmaps demonstrating intra-lesion heterogeneity consistent with malignancy; Figure S3: Photographs of three benign rectal lesions with 2D center of mass heatmaps demonstrating intra-lesion homogeneity.
