# Supplementary material for: Geotemporal Fluorophore Biodistribution Mapping of Colorectal Cancer: Micro and Macroscopic Insights
Source: Curr Oncol. 2024 Feb 2;31(2):849–61. doi: 10.3390/curroncol31020063 (PMC10887825; doi:10.3390/curroncol31020063)

Supplementary Figure S1. Analysis of mean and standard deviation of pixel intensities within lesions. Lesions are outlined using white light image for accurate delineation followed by conversion to greyscale and pixel analysis.

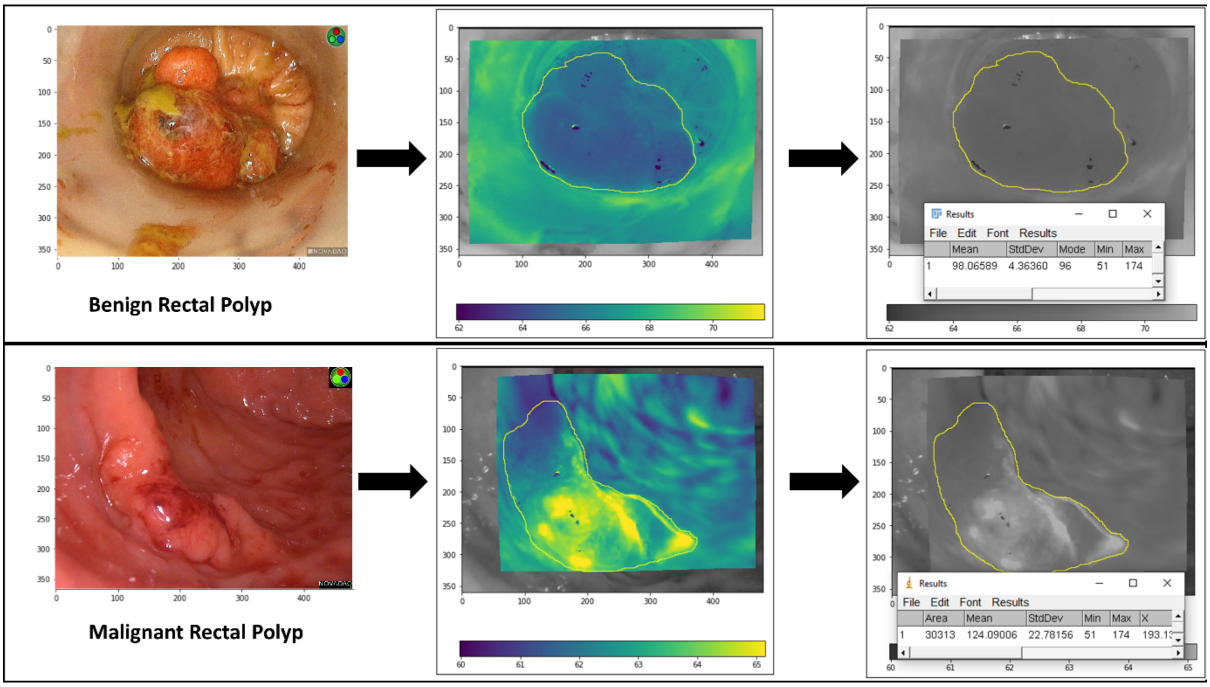

Supplementary Figure S2. Photographs of three malignant rectal lesions with 2D center of mass and outflow slope heatmaps demonstrating intra-lesion heterogeneity consistent with malignancy.

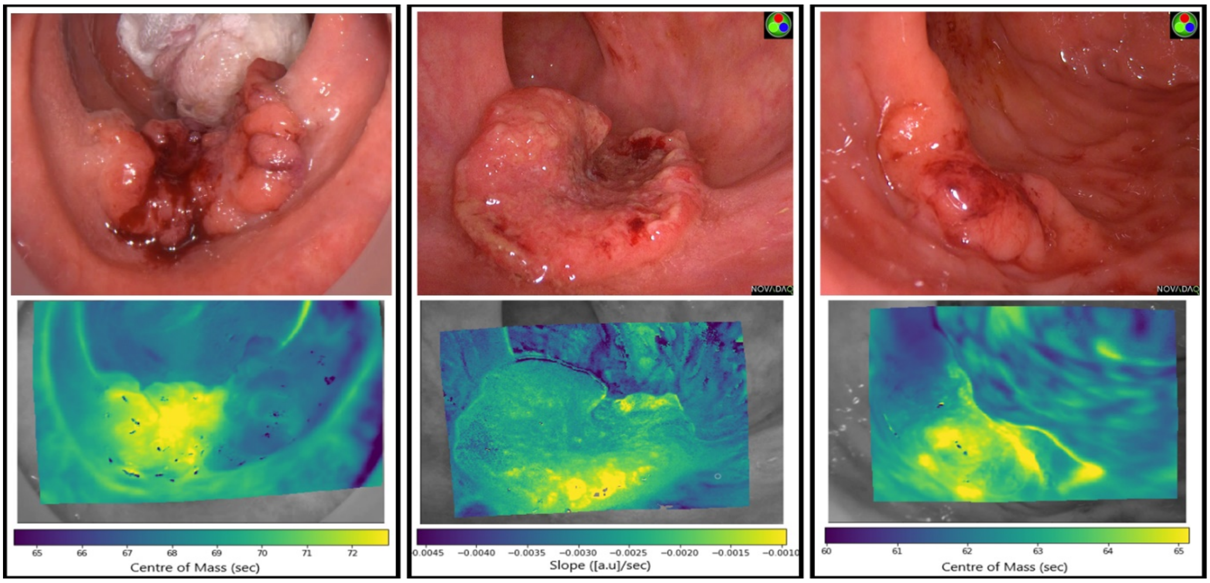

Supplementary Figure S3. Photographs of three benign rectal lesions with 2D center of mass heatmaps demonstrating intra-lesion homogeneity.

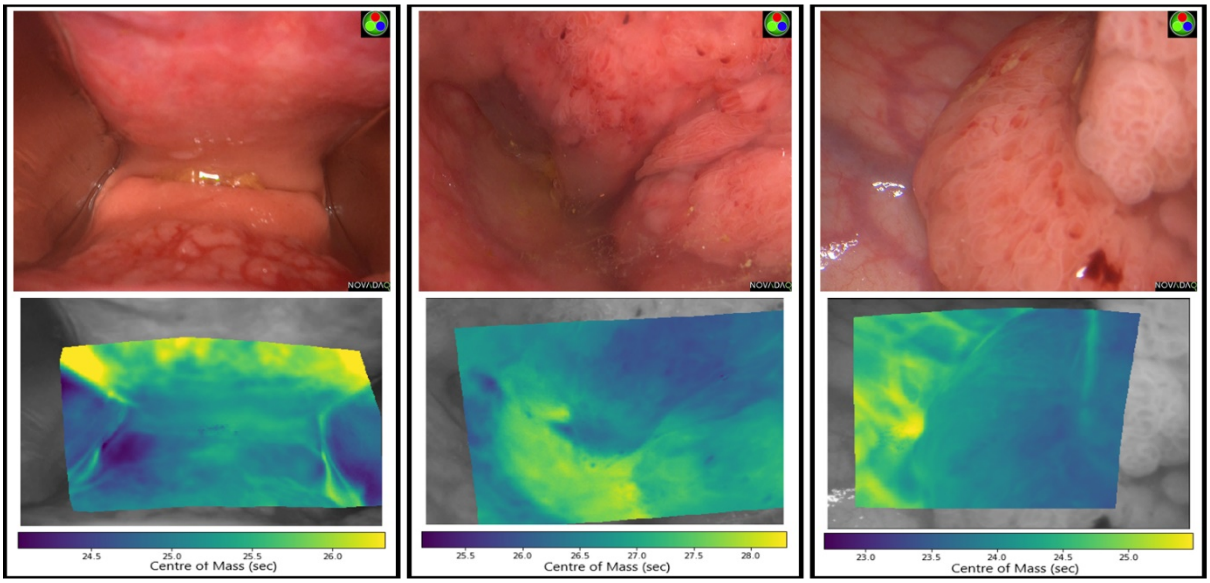

Supplement: Supplementary file 1 [file curroncol-31-00063-s001.zip › curroncol-2815162-supplementary.pdf]
